# National diagnostic reference levels for digital diagnostic and screening mammography in Uganda

**DOI:** 10.1371/journal.pone.0294541

**Published:** 2024-08-29

**Authors:** Denish Odongo, Alen Musisi, Richard Omara Okello, Felix Bongomin, Geoffrey Erem

**Affiliations:** 1 Department of Radiology, School of Medicine, Makerere University, Kampala, Uganda; 2 Department of Microbiology and Immunology, Faculty of Medicine, Gulu University, Gulu, Uganda; 3 Department of Radiology, St Francis Hospital Nsambya, Kampala, Uganda; University of Pisa, ITALY

## Abstract

**Introduction:**

Screening and diagnostic mammography are associated with some risk of radiation-induced breast cancer. This study was conducted to establish the National Diagnostic Reference Levels (NDRLs) for digital diagnostic and screening mammography in Uganda to achieve breast radiation dose optimization.

**Methods:**

A cross-sectional study was conducted among female participants recruited by consecutive sampling from three selected hospitals with digital mammography in Uganda. The study variables extracted from the mammography machines were exposure factors, compressed breast thickness (CBT), and Average Glandular Dose (AGD) of two standard mammogram views. The stratified National DRL was derived by calculating the 75^th^ percentile of the AGD across all the samples at various CBT ranges for both screening and diagnostic mammography in craniocaudal (CC) and mediolateral oblique (MLO) views.

**Results:**

We included 300 participants with mean ages of 50.28±9.32 and 47.45±13.45 years for the screening and diagnostic mammography, respectively. There were statistically significant positive correlations between AGD and exposure factors (mAs, kVp) (all p-values<0.0001). For screening mammography, mAs demonstrated a strong positive correlation (r = 0.8369 in CC, 0.8133 in MLO), whereas kVp showed a positive correlation with relatively lower coefficients (r = 0.3700 in CC, 0.3080 in MLO). For diagnostic mammography, mAs exhibited an even stronger positive correlation (r = 0.8987 in CC, 0.8762 in MLO), and kVp maintained a positive correlation with somewhat lower coefficients (r = 0.4954 in CC, 0.3597 in MLO). In screening mammography, for CBT within the range of (7–39) mm, the NDRLs were (1.5mGy, 1.66mGy) in CC) and MLO views. For CBT in the range of (40–59) mm, the NDRLs were (1.78mGy, 1.87mGy), and for CBT in the range of (60–99) mm, the NDRLs were (2.18mGy, 2.22mGy). For diagnostic mammography, the NDRLs were established as (1.7mGy, 1.91mGy), (2.00mGy, 2.09mGy), and (2.63mGy, 2.81mGy) for CBT ranges of (7–39) mm, (40–59) mm, and (60–99) mm, respectively.

**Conclusion:**

The NDRLs for digital screening and diagnostic mammography in Uganda have been proposed for the first time. The NDRL values in mammography should be specific to CBT ranges and mammographic views for both diagnostic and screening mammography.

## Introduction

According to the World Health Organization (WHO), approximately 2.26 million new cases of breast cancer in women were diagnosed globally in 2020, accounting for one in every eight cancer patients, making it the most prevalent cancer and the leading cause of cancer-related death among women [1]. However, this figure varies by region, for example, lung cancer was the leading cause of death in China though breast cancer was still the most prevalent cancer in that country in 2020 [2].

Modalities such as ultrasound and MRI can be used for breast examinations, but mammography is accepted to be the gold standard technique in breast cancer diagnostic studies [3]. The use of mammography has been shown to reduce deaths from breast cancer by 20–40% [4,5]. Mammography is performed using either the screen film mammography (SFM) or digital mammography machine for screening in asymptomatic women at increased risk of breast cancer, and diagnosis in women with symptoms suggestive of breast cancer [6].

In digital mammography, the x-rays transmitted through the breast are absorbed by an electronic detector, recorded, and displayed using computer image post-processing as opposed to SFM [7].

One of the serious risks associated with mammography is radiation-induced breast cancers. This is because a low dose of irradiation causes DNA damage in mammary epithelial cells following repeated mammographic imaging [8]. According to the reviewed International Commission on Radiological Protection (ICRP) of 2007, breast tissue has a tissue weighting factor of 0.12 which makes it one of the most radiosensitive tissues in the body with an increased risk of the stochastic effect of radiation [9].

The probability of occurrence of radiation-induced breast cancer increases with increasing average glandular dose (AGD) [10].

The Diagnostic Reference Level (DRL) was first introduced by ICRP in 1996 to optimize the dose for all ionizing radiation including mammography [11]. The DRL was purposefully introduced to help control the high radiation dose which increases the risk of radiation-induced breast cancer in women [12].

DRL is the Commission’s term for a form of investigation level used to aid in the optimization of protection in the medical exposure of patients for diagnostic and interventional procedures [13]. The DRL has a DRL quantity (which is a commonly and easily measured or determined radiation metric that assesses the amount of ionizing radiation used to perform a medical imaging task) and DRL value (an arbitrary notional value of a DRL quantity, set at the 75th percentile of the distribution of the medians of distributions of the DRL quantity obtained from surveys or other means). The DRL quantity used in mammography is the Glandular dose (GD) also known as the average glandular dose. It is appropriate to use GD to establish DRL quantity in mammography even though it measures the organ dose rather than the amount of ionizing radiation used to perform the mammography. This is due to the large variability of entrance-surface air kerma (K_a,e_) and incident air kerma (K_a,i_) with kV and different anode/filter combinations, even for the same breast thickness.

There is no established national or local DRL for digital mammography in Uganda despite having 18 mammography machines currently registered with the Atomic Energy Council (Only five of these eighteen are digital mammographic machines). We aimed to establish the national DRLs (NDRLs) for both screening and diagnostic mammography in Uganda as well as establish the relationship between the AGD and the radiation exposure factors.

## Materials and methods

### Study design

This was a cross-sectional study conducted for 2 years from the 1^st^ of January 2021 to the 31^st^ of December 2022.

### Study setting

The purposive sampling method was used to select three (60%) of the 5 digital mammography machines in Uganda. The ICRP 135 requires 35–50% for the initial establishment of the national DRL [11]. These hospitals were kept anonymous for confidentiality by assigning the codes A, B, and C. Two of these hospitals were located in Kampala and one was located in Jinja City. There was no functional digital mammography machine in the government hospital during this study period.

### Sample size estimation

According to the ICRP 135 of 2017 updated in 2019, data on DRL quantities in mammography requires a minimum of 50 patients per mammography machine [11]. Data from 50 participants were collected for each of the 3 study hospitals per indication (screening and diagnostic mammography). The total number of participants for this study was (50x3x2) = 300. For each participant, 4 breast mammographic views (RMLO, LMLO, RCC, and LCC) were considered. Therefore, the total number of mammographic views (images) for this study was (4x300) = 1200.

### Sampling method

A consecutive sampling method was used to select records of 50 females who underwent screening and 50 females for diagnostic mammography from hospitals **A, B** and **C** and a total of 300 participants were selected.

### Data tool and data collection

The information about the machine model, manufacturers, year of manufacturing, and year of installation was recorded in the data abstraction form. The study variables for the mammographic images of the left craniocaudal (LCC), right craniocaudal (RCC), left mediolateral oblique (LMLO), and right mediolateral oblique (RMLO) were retrospectively accessed from 25^th^ April-2023 to 25^th^ May-2023 and extracted from the mammography Digital Imaging and Communications in Medicine (DICOM) header, then recorded in a data abstraction form. The Image quality was graded using the PGMI (Perfect, Good, Moderate, and Inadequate) method for evaluation of the clinical image quality in mammography [14]. The most qualified and experienced radiographers from every centre were assigned to evaluate the image quality, categorize them into PGMI [15] and record them in a data collection tool.

### Study variables

The study variables in this study were the age of patients (years), kilovoltage peak (kVp), milliampere-seconds (mAs), breast compression force (BCF), compressed breast thickness (CBT) in millimetres (mm), anode target/filter combinations, entrance surface dose (ESD) in milli Gray (mGy) and AGD in mGy. In this study, the AGD and ESD for each acquired image were calculated automatically by the mammographic machine and recorded in the system using the methods described by Dance et al. [16–18].

### Data management and analysis

The data from the data collection tool were entered manually into the Excel sheet. The AGD, CBT, BCF, ESD, mAs, and kVp of the right craniocaudal (RCC) and left craniocaudal (LCC) views of the breast images were summed up and divided by two to obtain the mean for the craniocaudal (CC) view. A similar process was repeated for the mediolateral oblique (MLO) view. These were then exported to STATA version 17 software and analyzed. Descriptive statistics were used to obtain the mean, median, standard deviation, percentiles, and range values. AGD’s relationship with the exposure factors (kVp, mAs), ESD, CBT, and BCF was tested using a Pearson correlation coefficient test and presented in table form. The values of the CBT were grouped into three categories of a narrow range which were representative of CBT for the Ugandan women population as recommended by ICRP number 135 for every country to determine their women’s CBT ranges [11,19].

The NDRL values of the screening and diagnostic mammography were determined by calculating the 75^th^ percentile of the median values of the AGD across the identified CBT ranges in each mammographic view (CC and MLO) from the combined data across all the 3 hospitals. A comparison of established NDRL in this study with the NDRLs of other countries was made and presented in a table.

### Quality assurance and quality control

Quality control tests were performed by a qualified medical physicist and radiographer for all the 3 mammography machines. These tests were daily digital tests with monitor checks, weekly digital tests requiring homogeneity (image quality) checks, weekly checks of automatic exposure control, and 3–6 monthly tests, performed by a medical physicist, which include kVp accuracy and reproducibility, and output linearity. The accuracy of the AGD registered by the mammography machines was not established since all three hospitals in this study lacked the mammographic x-ray filter as an accessory required for establishing the half value layer (HVL) necessary in the calculation of the AGD.

The research assistants were adequately trained and routinely supervised by the principal investigator to ensure the correct use of the data collection tool and adherence to ethical principles. The completed abstraction forms were checked and verified with the data from the machine for completeness and accuracy.

### Ethical considerations

Ethical approval to conduct the study was obtained from the Institutional Review Board (IRB) of the School of Medicine, Makerere University College of health sciences (protocol reference number: Mak-SOMREC-2023-562). The IRB also provided a waiver of consent by the participant since data collection was retrospective. Permission to access patients’ data and their mammographic images was obtained from the executive directors of the respective hospitals which provided the mammographic services. All the information and mammograms were kept confidential and used only for the present study by restricting access to the study information which was in hard copy as well as password protection of the soft copy documents. Anonymity was maintained for both the participating hospitals and study participants by using alphabetical letters and serial numbers for hospitals and patients rather than their names respectively.

## Results

In this study, three (3) out of the five (5) digital mammography machines were selected from three Hospitals A, B, and C, accounting for 60% of all digital mammography machines in Uganda.

### Results on mammography machines for hospitals A, B, and C

Hospitals A and C had Senographe digital mammography machines with similar voltage (220-230V) and frequencies (50/60Hz). Their power inputs were 6.9kVA and 7kVA respectively.

Hospital B had a Siemens digital mammography machine. Its voltage is 220-230V, frequency of 50/60Hz, and power input of 7.5kVA. All hospitals in this study used a large focal spot size (0.3mm) and tungsten/Rhodium (W/Rh) anode target/filter combination to acquire mammographic images for both screening and diagnostic mammography (Table 1).

**Table 1:** The Digital mammography machine from hospitals A, B and C.

Mammography Machine parameters	Hospitals		
	A	B	C
**Ownership**	Private	PNFP	Private
**Type**	DR	DR	DR
**Model/make**	Senographe Crystal Nova	Mammomat inspiration Prime	Senographe, Crystal Nova
**Manufacturer**	GE Medical System, China	Siemens Health Care, Germany	GE Medical System Korea
**Year of manufacture**	June/2020	October/2019	December/2018
**Year of installation**	2021	2020	2020
**Focal spot size used**	0.3 mm	0.3 mm	0.3 mm
**Anode/filter combination used**	Tungsten/Rhodium (W/Rh)	Tungsten/Rhodium (W/Rh	Tungsten/Rhodium (W/Rh

Where, **DR** = Digital Radiography, **GE** = General Electric; **PNFP**–private not for profit.

### Demography of the study participants

In Table 2, the age range of the participants for screening mammography was 33–76 years with a mean age of 50.28 ± 9.32 SD. For the diagnostic mammography, the age range was 15–84 years with a mean age of 47.45 ±13.45 SD.

**Table 2:** The number of participants under 30 years, 30–44 years, and ≥45 years of age (N = 300).

Age (years)	Screening Mammography (n = 150)	Diagnostic Mammography (n = 150)
<30	0 (0%)	6 (4%)
30 to 44	44(29.33%)	64 (42.67%)
≥45	106 (70.67%)	80(53.33%)
**Total**	**150**	**150**
**The mean, standard deviation and ranges of the age of the participants**		
Mean±SD	50.28±9.32	47.45±13.45
Range	33–76	15–84

Source: Data from hospitals A, B, and C (N = 150 Screening and 150 diagnostics, total = 300).

### The relationship of AGD with the kVp, mAs, CBT, BCF, and ESD

The relationship of the AGD with mammographic radiation exposure factors (kVp and mAs), CBT, BCF, and Entrance Surface Dose (ESD) was assessed using the Pearson correlation coefficient test (Table 3).

**Table 3:** Pearson correlation coefficient for the relationship between the AGD with kVp, mAs, ESD, CBT, and BCF.

Modality	Variable	Projections	Pearson correlation coefficient (r)	95% CI	p-value
**Screening**	KVp	CC	0.3700	0.223, 0.501	<0.0001*
		MLO	0.3080	0.155, 0.446	<0.0001*
	mAs	CC	0.8369	0.781, 0.879	<0.0001*
		MLO	0.8133	0.751, 0.861	<0.0001*
	ESD	CC	0.8860	0.846, 0.916	<0.0001*
		MLO	0.6689	0.570, 0.749	<0.0001*
	CBT	CC	0.3783	0.232, 0.508	<0.0001*
		MLO	0.4437	0.305, 0.564	<0.0001*
	BCF	CC	-0.1993	-0.348, -0.040	0.0145*
		MLO	-0.0935	-0.250, 0.068	0.2552
**Diagnostic mammography**	kVp	CC	0.4954	0.364, 0.607	<0.0001*
		MLO	0.3597	0.212, 0.492	<0.0001*
	mAs	CC	0.8987	0.863, 0.926	<0.0001*
		MLO	0.8762	0.833, 0.909	<0.0001*
	ESD	CC	0.9168	0.887, 0.939)	<0.0001*
		MLO	0.9063	0.873, 0.931	<0.0001*
	CBT	CC	0.3932	0.249, 0.521	<0.0001*
		MLO	0.3857	0.240, 0.514	<0.0001*
	BCF	CC	0.0598	-0.101, 0.218	0.4676
		MLO	0.0220	-0.139, 0.182	0.7894

There was a strong positive correlation between AGD and mAs in both views of the screening mammography with r = 0.8369 and 0.8133 (all p-values <0.0001) in both CC and MLO projections. For diagnostic mammography, the r = 0.8987 and 0.8762 (all p-values <0.0001) in both CC and MLO projections. A strong positive correlation was seen with ESD too. The rest of the exposure factors showed a moderate positive correlation with the AGD for both modalities and mammographic views except for BCF which showed a negative correlation with the AGD in the CC mammographic view in the screening mammography (r = -0.1993, p = 0.0145).

### Description of the digital mammographic variables among hospitals

In Table 4, the mAs range and means for hospital B were comparable to those of hospital A for both screening and diagnostic mammography and views. The mAs range and mean values for hospital C were higher than hospitals A and B in the CC and MLO views for both screening and diagnostic mammography. Hospital C had the least BCF. The range of BCF (N) in the CC and MLO views was 30-150N for both the screening and diagnostic mammography. However, the range of BCF (N) was 35-150N for diagnostic mammography in a CC view. The mean, median, minimum, and maximum ranges of ESD for hospital C were higher than those in hospitals A, and B.

**Table 4:** Mean, Median, SD, range of mAS, BCF, and ESD for Hospital A, B, C, and ALL.

Hospital	Views	Modality	mAs		BCF (N)		ESD (mGy)	
			Mean ±SD	Range	Mean±SD	Range	Mean±SD	Range
**A**	CC	Screening	91.84 ±30.07	36.95–134.	98.1±33.65	30–170	4.68±1.9	1.2–8.69
		Diagnostic	115.0 1±43.03	30.15–218	133.7±48.1	45–200	6.22±3.0	1.05–13.49
	MLO	Screening	105.88 ±39.85	37.1–221	114±40.18	50–200	6.21±4.9	0.96–6.24
		Diagnostic	126.73 ±46.83	36.8–250	145.9±41.2	35–200	7.03±3.4	1.37–15.11
**B**	CC	Screening	83.09±24.61	37.2–130.05	97.41±14.8	58–133.5	3.84±2.1	1.25–11.95
		Diagnostic	91.24.01±38	40.25–212.65	95.58±9.58	79–135.5	3.55±1.4	0.85–6.55
	MLO	Screening	103.59±37.78	27.85–206.4	106.47±22	77–165	4.75±2.0	0.7–10.8
		Diagnostic	109.97±31.61	43.75–172.55	100.33±18	72.5–171	5.17±2.5	1.35–15.95
**C**	CC	Screening	129.35±36.16	73.8–155.7	78.8±30.04	30–150	6.22±2.0	2.24–8.49
		Diagnostic	91.24.01±38	40.25–212.65	78±39.45	30–150	6.62±2.3	3.02–13.61
	MLO	Screening	143.30±46.24	60.5–192.5	88.1±31.83	35–150	6.92±2.7	2.56–13.85
		Diagnostic	109.97±31.61	43.75–172.55	84.02±39.7	30–150	7.68±3.0	3.04–15.26
**ALL**	CC	Screening	98.29±34.26	36.95–184	91.44±28.68	30–170	4.81±2.1	0.85–10.72
		Diagnostic	111.86±43.09	30.15–252.9	102.43±43.0	30–200	5.56±2.8	1.05–13.49
	MLO	Screening	113.43±40.92	27.85–222.5	102.86±33.8	35–200	5.96±3.5	0.7–6.4
		Diagnostic	26.67±44.04	36.8–253	110.08±43.3	30–200	6.63±3.1	1.35–16.48

Meanwhile, the overall mean values for ESD were lower in CC and higher in MLO for both screening and diagnostic mammography. This trend was not seen in the overall mAs and BCF.

### The national DRLs and kVp at various CBT ranges for the screening mammography

In Table 5, the national DRL values at stratified CBT ranges per mammographic views for digital screening mammography were computed as the 75^th^ percentile/3^rd^ quartile of the median values of the AGD of all samples across all the hospitals. The national DRLs at CBT ranges of (7–39) mm, (40–59) mm, and (60–99) mm were (1.5, 1.66) mGy, (1.78, 1.87) mGy, and (2.18, 2.22) mGy for CC and MLO views respectively. In the same Table 5, the mean CBT was higher in MLO than in CC view for all the categories of CBT ranges. The mean kVp increased with CBT and was higher in MLO than in CC views. The range of the AGD was (0.45–3.27) mGy.

**Table 5:** The mean, standard deviation, and range of kVp and AGD plus the 75^th^ percentile of median values of AGD at various CBT ranges for all the participants.

Modality	CBT Range	View	NO_ of images	CBT (mm) Mean±SD	kVp		AGD			
					Mean±SD	Range	Mean ±SD	Median	Range	75^th^ percentile (NDRL)
**Screening**	7–39	CC	23	28.34±8	27.41 ±0.69	26–29	1.23±0.40	1.28	0.51–2.01	**1.54**
		MLO	19	28.95±7	27.62 ±0.70	25.5–28	1.32±0.43	1.31	0.45–1.89	**1.66**
	40–59	CC	91	49.49±5	28.64 ±0.46	27.5–29	1.44±0.52	1.51	0.53–2.81	**1.78**
		MLO	70	50.59±4	28.69 ±0.46	27.5–30	1.54±0.50	1.58	0.72–2.7	**1.87**
	60–99	CC	36	66.31±4	29.74 ±1.04	29–34.5	1.76±0.58	1.87	0.98–2.74	**2.18**
		MLO	61	69.52±8	29.82 ±0.70	28.5–32	1.85±0.66	1.78	0.6–3.27	**2.22**
**Diagnostic**	7–39	CC	23	31.43±7	27.67 ±0.76	26–29	1.38±0.53	1.43	0.55–2.64	**1.7**
		MLO	16	31.31±7	27.88±0.85	26.5–29	1.52±0.70	1.45	0.59–3.05	**1.91**
	40–59	CC	90	49.57±5	28.66±0.48	27.5–29	1.61±0.64	1.59	0.73–3.23	**2.00**
		MLO	78	51.19±5	28.82±0.53	27.5–30	1.69±0.62	1.68	0.68–3.47	**2.09**
	60–99	CC	37	66.49±5.	29.61±0.49	29–31	2.04±0.74	2.13	0.95–3.55	**2.63**
		MLO	56	70.04±7	29.96±0.77	29–32	2.16±0.74	2.22	1.09–3.81	**2.81**

*p<0.05.

### The national DRLs and kVp at various CBT ranges for diagnostic mammography

Similar to that of screening mammography, the national DRLs for diagnostic mammography at CBT ranges of (7–39) mm, (40–59) mm and (60–99) mm were (1.7, 1.91) mGy, (2.00, 2.09) mGy, and (2.63, 2.81) mGy respectively. The national DRL values for diagnostic mammography were also higher than those for screening mammography for the corresponding CBT and mammographic views.

The trend of the CBT and kVp were similar but had higher values than those in the screening mammography of the corresponding CBT and mammographic views (Table 5).

### Image quality assessment

The PGMI (Perfect, Good, Moderate and Inadequate) method for evaluation of the clinical image quality in mammography [14] was used. The overall result indicated that more than 50% of all the images in both views were classified as perfect (74% and 57.33% for CC and MLO views respectively). However, 3.33% of the images in MLO views were graded as inadequate (Table 6).

**Table 6:** Image quality assessment.

Grades of image quality		P		G		M		I		TOTAL	
Hospital	Views	Pts NO:-	%age	Pts NO:-	%age	Pts NO:-	%age	Pts NO:-	%age	Pts NO:-	%age
**A**	CC	28	**56**	2	**4**	19	**38**	1	**1**	50	**100**
	MLO	21	**42**	1	**2**	27	**54**	1	**2**	50	**100**
**B**	CC	39	**78**	2	**4**	9	**18**	0	**0**	50	**100**
	MLO	29	**58**	5	**10**	15	**30**	1	**2**	50	**100**
**C**	CC	44	**88**	2	**4**	3	**6**	1	**2**	50	**100**
	MLO	36	**72**	1	**2**	10	**20**	3	**6**	50	**100**
**ALL**	CC	111	**74**	6	**4**	31	**20.66**	2	**1.33**	150	**100**
	MLO	86	**57.33**	7	**4.66**	52	**34.66**	5	**3.33**	150	**100**

### Comparison of the NDRL (this study) with other country’s established NDRLs

Different countries set their NDRLs for variable CBT as shown in Table 7. The NDRL/woman for Malaysia was set for CBT ranges of (20–39, 40–59, and 60-99mm) [19], similar to the one in the current study (7–39, 40–59, and 60-99mm). The respective NDRL values for these two countries were 1.13, 1.52, and 1.87mGy for Malaysia [19] and 1.57, 1.78, and 2.34mGy for Ugandan screening mammography. The national NDRL/woman for diagnostic mammography in this study (1.8, 2.05, and 2.57mGy) was higher than those for screening mammography in Malaysia for similar CBT ranges [19].

**Table 7:** Comparison of this study’s NDRLs with other country’s established NDRLs.

Source of data/country	NO:—women	CBT (mm)	NDRL/view		NDRL/woman
			CC	MLO	
**The current study (Uganda); Screening**	**150**	**7–39**	**1.5**	**1.66**	**1.57**
		**40–59**	**1.78**	**1.87**	**1.78**
		**60–99**	**2.18**	**2.22**	**2.34**
**The current study (Uganda); Diagnostic**	**150**	**7–39**	**1.7**	**1.91**	**1.83**
		**40–59**	**2.0**	**2.09**	**2.05**
		**60–99**	**2.63**	**2.81**	**2.57**
O’Leary et al [20], Ireland	1010	40–68	1.45	1.56	---
Karsh et al [21], Gaza, Palestine	200	60–70	1.15	1.24	1.18
Suleiman et al [22], Australia	----	30–40	---	--	1.31
		40–45	---	--	1.5
		45–55	---	--	1.67
		55–60	---	--	2.06
Mohd et al [19], Malaysia	87	20–39	---	--	1.13
		40–59	--	--	1.52
		60–99	--	--	2.87
Dzidzornu et al [23], Ghana	979	3–100	1.6	2.4	---
		55–60	2.0	2.5	---

## Discussion

DRL is the term used by the International Commission of Radiation Protection to mean a form of investigation level used to aid in the optimization of protection in the medical exposure of patients for diagnostic and interventional procedures [13]. This study determined and compared the NDRLs for digital mammography with those of other countries using data from 60% of the total digital mammography machines in Uganda.

The digital mammography machines in 2/3 of the hospitals were Senographe, and Siemens in 1/3^rd^ of hospitals in Uganda.

All hospitals in this study used a large focal spot size (0.3 mm) and a tungsten/rhodium (W/Rh) anode target/filter combination to acquire mammography images both for screening and diagnostic mammography. Modern mammography systems most frequently use a nominal focal spot size of 0.3 mm for regular mammography and 0.1 mm for magnification procedures [24]

The mean age of the patients recorded in this study for the screening and diagnostic mammography (50.28±9.32 and 47.45±13.45 years respectively) was slightly lower than the mean ages in other countries including Ghana {(54 ± 10.0 years) [25]}, {(56 ± 10.0 years) [26]} and Australia {(60 ± 7.9 years) [22]. Furthermore, the patients for screening were much younger than those for diagnostic mammography with age ranges of 15–84 years and 33–76 years respectively. The young age of the patients for the diagnostic mammography was probably because of the younger age of distribution of breast cancer in Uganda [27]. However, 15 years of age was below the recommended minimal age (25 years) for performing mammography in symptomatic patients in Uganda [28].

This study found a strong positive relationship between AGD and some exposure parameters. The relationships were comparable in both views of screening and diagnostic mammography. The findings recorded are in agreement with a report by Ko et al [29] which indicated mAs, and kVp correlated positively with CBT.

The National DRL values for diagnostic mammography obtained in this study were higher than those for screening mammography for the corresponding CBT ranges and mammographic views. This could be explained by the younger women who underwent diagnostic mammography in this study. The younger women have a greater proportion of breast glandular tissue than fatty tissue, requiring higher x–rays exposure, hence higher DRL values for diagnostic mammography than screening mammography. This finding is consistent with a study by O’Leary & Rainford (Screening service DRL at 55–65 mm CBT: 1.75 mGy (screening) vs. 2.4 mGy (symptomatic) [30].

The national DRL in the current study was comparable with those in Malaysia [19]. However, most countries set their DRLs for a single narrow CBT range as in Malta which was set at a CBT range of 50-70mm, while Sudan [31], and Ireland [32] set their national DRLs at 55-65mm CBT range. The comparison of established national DRLs in this study with those of other countries is therefore challenging due to the lack of a standard CBT range used for setting the DRLs.

The overall image quality of more than 50% was classified as perfect (74% and 57.33% for CC and MLO projections respectively) and less than 3% (1.33%) was inadequate for a CC projection. One of the hospitals had an inadequate image quality of up to 6%. By comparison, 6.2% were inadequate according to O’Leary et al in Ireland [20]. To effectively use PGMI, a minimum of 50% of audits of 50 randomly selected cases graded as P or G categories (75% desirable), P or G or M categories (97% desirable) and the repeat rate should be less than 3% of consecutive images to be classified as "Inadequate". In this study, the mammograms with image quality classified as “inadequate” were excluded.

## Conclusion

There was a positive relationship between the AGD and exposure factors (mAs, kVp), and ESD. The patient-based NDRL values for digital screening and diagnostic mammography at various ranges of compressed breast thickness in Uganda have been proposed for the first time providing valuable insights into the radiation dose status.

The established NDRLs in this study were comparable to the NDRL values for a few countries that set their NDRLs at a similar compressed breast thickness.

### Recommendation

The NDRL values in mammography should be specific to compressed breast thickness ranges, mammographic views, and indications for mammography (screening and diagnostic mammography) as significant variations in their DRL values were shown in this study.

### Study limitation

The major limitation of this study is the unknown accuracy of the AGD recorded by the mammographic machines we collected the data from. The physical calculation of the AGD was not done due to a lack of a mammographic x-ray filter accessory needed to measure the half-value layer (HVL) required to calculate the AGD as part of quality assurance.

## Supporting information

S1 Appendix PGMI image quality for mammography. (PDF)

S2 Appendix Raw data set for NDRLs for mammography in Uganda. (XLSX)
