# Supplementary material for: National diagnostic reference levels for digital diagnostic and screening mammography in Uganda
Source: PLoS One. 2024 Aug 29;19(8):e0294541. doi: 10.1371/journal.pone.0294541 (PMC11361431; doi:10.1371/journal.pone.0294541)
Supplement: S1 Appendix — (PDF) [file pone.0294541.s001.pdf]

S1 appendix: PGMI _Image quality for mammography

PGMI assessment grading form (Tick only one appropriate grade **P, G, M** and **I** for each mammographic view for each patient**.** Use the PGMI guide for reference**).**

Patient's serial Number_________Hospital: A ☐ **B** ☐ **C** ☐

	**PGMI Grades**			
**Mammogram view**	**P**	**G**	**M**	**I**
**CC**				
**MLO**				

Where, P= **Perfect**, G=**Good**, M=**Moderate** and I=**Inadequate**.

PGMI GUIDE

The PGMI 9 criteria for mammography image quality assessment method (Moreira et al., 2005)

1. All breast tissue imaged (fat tissue visualized posterior to the glandular tissue)
2. The correct image identifications are clearly shown
3. There is correct exposure
4. Good compression
5. Absence of movement
6. Correct processing
7. No artefacts
8. No skin folds seen
9. Both images are symmetrical

Criterion 1 varies for CC and MLO views as below.

Criterion 1-Specific for MLO view.

1. All breast tissue imaged:

- Pectoral muscle shadow to nipple level
- Full width of the pectoral muscle
- Nipple in profile (retro-areolar tissue well separated)
- Infra-mammary fold well demonstrated
- Posterior nipple line (PNL) within 1 cm of PNL on CC view

Criterion 1-Specific to the CC view.

1. All breast tissue imaged
   - medial border well demonstrated
   - nipple in profile (retro-areolar tissue well separated)
   - nipple in the midline of imaged breast
   - posterior nipple line (PNL) within 1 cm of PNL on MLO view

Table summarizing the key in PGMI grading of image quality in mammography.

	**Grading**			
**View**	**P (Perfect)**	**G (Good images)**	**M (Moderate images)**	**I (Inadiquate images)**
**CC**	• Images meet criteria 1−9	• 1-Criterion 1 is met for the CC view<br>• 2-6-Images meet criteria for image assessment 2−6 for CC view.<br>• 7-9. Images displaying minor degrees of variation in criteria 7, 8 and 9 for CC view.	• 1-Most breast tissue imaged (nipple not in profile but is clearly distinguishable from retro-areolar tissue, nipple not in the midline (significant bias)<br>• 2-correct(ed) image identification<br>• 3-correct exposure<br>• 4-adequate compression<br>• 5-absence of movement<br>• 6-correct processing<br>• 7-artefacts which do not obscure the image<br>• 8-skin folds which do not obscure the breast tissue<br>• 9-asymmetrical image	• 1-A significant part of the breast was not imaged<br>• 2-incomplete or incorrect identification<br>• 3-incorrect exposure<br>• 4-inadequate compression hindering diagnosis<br>• 5-blurred image<br>• 6-incorrect processing<br>• 7-overlying artefacts<br>• 8-skin folds obscuring the image
MLO	• Images meet criteria for	• All breast tissue	• 1-most breast tissue imaged. (pectoral muscle not to nipple level but	• 1-a significant part of the breast not imaged

	image assessment 1−9 for MLO	imaged for criterion 1.<br>• **2- 6**. images meet criteria 2−6 for MLO<br>• **7 - 9.** MLO images displaying minor degrees of variation in criteria 7-9 for MLO	posterior breast tissue adequately shown, nipple not in profile but clearly distinguishable from retro-areolar tissue, IMF not clearly demonstrated but breast tissue adequately shown)<br>• 2-correct(ed) image identification<br>• 3-correct exposure<br>• 4-adequate compression<br>• 5-absence of movement<br>• 6-correct processing<br>• 7-artefacts which do not obscure the image<br>• 8-skin folds which do not obscure the breast tissue<br>• 9-asymmetrical images	• 2-incomplete or incorrect identification<br>• 3-incorrect exposure<br>• 4-inadequate compression hindering diagnosis<br>• 5-blurred image<br>• 6-incorrect processing<br>• 7-overlying artifacts<br>• 8-skin folds obscuring the image
